# A cross-sectional comparison of gut metagenomes between dairy workers and community controls

**DOI:** 10.1186/s12864-024-10562-1

**Published:** 2024-07-20

**Authors:** Pauline Trinh, Sarah Teichman, Marilyn C. Roberts, Peter M. Rabinowitz, Amy D. Willis

**Affiliations:** 1 Department of Biostatistics, University of Washington, Seattle, USA; 2 Department of Statistics, University of Washington, Seattle, USA; 3 Department of Environmental and Occupational Health Sciences, University of Washington, Seattle, USA

**Keywords:** Shotgun metagenomics, Livestock farming, Antibiotic resistance, Microbiome, Virulence factors

## Abstract

**Background:**

As a nexus of routine antibiotic use and zoonotic pathogen presence, the livestock farming environment is a potential hotspot for the emergence of zoonotic diseases and antibiotic resistant bacteria. Livestock can further facilitate disease transmission by serving as intermediary hosts for pathogens before a spillover event. In light of this, we aimed to characterize the microbiomes and resistomes of dairy workers, whose exposure to the livestock farming environment places them at risk for facilitating community transmission of antibiotic resistant genes and emerging zoonotic diseases.

**Results:**

Using shotgun sequencing, we investigated differences in the taxonomy, diversity and gene presence of 10 dairy farm workers and 6 community controls’ gut metagenomes, contextualizing these samples with additional publicly available gut metagenomes. We found no significant differences in the prevalence of resistance genes, virulence factors, or taxonomic composition between the two groups. The lack of statistical significance may be attributed, in part, to the limited sample size of our study or the potential similarities in exposures between the dairy workers and community controls. We did, however, observe patterns warranting further investigation including greater abundance of tetracycline resistance genes and prevalence of cephamycin resistance genes as well as lower average gene diversity (even after accounting for differential sequencing depth) in dairy workers’ metagenomes. We also found evidence of commensal organism association with tetracycline resistance genes in both groups (including *Faecalibacterium prausnitzii*, *Ligilactobacillus animalis*, and *Simiaoa sunii*).

**Conclusions:**

This study highlights the utility of shotgun metagenomics in examining the microbiomes and resistomes of livestock workers, focusing on a cohort of dairy workers in the United States. While our study revealed no statistically significant differences between groups in taxonomy, diversity and gene presence, we observed patterns in antibiotic resistance gene abundance and prevalence that align with findings from previous studies of livestock workers in China and Europe. Our results lay the groundwork for future research involving larger cohorts of dairy and non-dairy workers to better understand the impact of occupational exposure to livestock farming on the microbiomes and resistomes of workers.

**Supplementary Information:**

The online version contains supplementary material available at 10.1186/s12864-024-10562-1.

## Background

Next-generation sequencing has facilitated the study of entire microbial communities of culturable and unculturable microorganisms, revealing the profound impact that the human gut microbiome has on immune homeostasis [1–3], disease development [4–7], and even resistance to pathogen invasion [8–11]. The human gut microbiota is influenced by both host genetics [12, 13] and environmental factors, including diet [14, 15], geography [16], and medications [17, 18]. Recent research suggests that environmental factors outweigh host genetics in shaping the gut microbiome [19, 20]. Consequently, environments that are rich in antibiotic resistant organisms, antibiotic residues, antibiotic resistance genes (ARGs), and/or zoonotic pathogens, such as livestock farms, may pose significant risks to public health, as these environments may serve as hotspots for antibiotic resistance and zoonotic disease emergence and propagation [21, 22]. Studies examining changes in the human microbiome and resistome in response to occupational exposure to livestock on farms may shed light on the potential risks of these environments for transmission and spread of zoonotic diseases and antibiotic resistance.

Modern farming practices and agricultural intensification have been linked to the emergence and amplification of zoonotic diseases and antimicrobial resistance (AMR), with livestock potentially serving as intermediate hosts for pathogens [23, 24]. Transmission of both zoonotic pathogens and antibiotic resistance genes can occur through direct or indirect contact at the human-animal interface, placing livestock workers and those in contact with these workers at risk of transmission and infection [25, 26]. Several shotgun metagenomic studies have looked at the effect of occupational exposure to animal agriculture on ARG carriage, finding higher prevalence of ARGs as well as evidence of transmission of ARGs from animal farming environments to workers [27–29]. While these studies highlight some potential impacts of exposure to ARG-rich animal farming environments, they either focused primarily on understanding the presence of ARGs in total community DNA without contextualizing ARGs to particular species of bacteria, or they used cultured isolates of a single bacterial species (e.g., *Escherichia coli*) to understand species-level antibiotic resistance transmission [27–29]. Furthermore, these studies did not examine virulence factor genes, which encode for functions that can cause disease and assist an organism with persisting within a host [30]. While virulence factors have historically been associated with pathogens [30] they have also been identified on commensal or non-pathogenic genomes [31, 32], and their transmission can occur between pathogens and commensals by mobile genetic elements transmission [33, 34].

To better understand the effect of the livestock farming environment on the human gut microbiome of workers — including virulence factors, taxonomic associations of ARGs, and the role of commensal organisms in ARG transmission — we compared dairy worker and community control gut microbiomes using shotgun metagenomic sequencing. We studied differences with respect to diversity, taxonomic composition, and the carriage of virulence factor and antibiotic resistant genes. We additionally evaluated potential taxonomic affiliations of genes conferring resistance to beta-lactams (cephamycin and cephalosporins) and tetracyclines through reconstruction of their genomic context, and assessed differences in taxonomic context based on group association.

## Materials and methods

### Study participant selection

We performed metagenomic sequencing on a subset of stool samples from participants in the Healthy Dairy Worker study. The Healthy Dairy Worker study is a prospective cohort study that focuses on the effects of dairy farm exposure on the fecal and nasal microbiome, and immune and respiratory function of dairy farm workers. The study began recruitment of subjects on a rolling basis in May 2017 and involves collection of fecal and nasal samples, as well as health history data on participants at baseline enrollment, 3, 6, 12, and 24 months. Dairy workers were recruited from 3 conventional large ($>5,000$ animals) farms in the Yakima Valley of Eastern Washington State and community controls were recruited from surrounding communities. Recruitment of both community controls and dairy workers was done through snowball sampling where research participants assisted in identifying other potential participants. Eligibility to be a participant as a dairy worker required subjects to have been working on a dairy farm for at least 6 months. Eligibility as a community control required participants to have no prior work experience on a dairy farm in the previous 5 years, to have not lived on a dairy farm, and to have no current household member who worked on a dairy farm in the previous 5 years. Participants were consented by bilingual study staff and received an incentive payment for enrollment and subsequent sampling. Participants were asked to participate in self-reported surveys collecting information on health and work history. Sample collection and study activities were approved by the University of Washington Institutional Review Board under STUDY00000042. Study protocols have been previously described [35].

To conduct the current cross-sectional metagenomics study, we selected shotgun sequencing data of 16 fecal samples (the maximum possible with budget constraints) taken from the Healthy Dairy Worker study cohort. These samples came from 10 dairy workers and 6 community controls, all sampled at baseline enrollment. We selected the 10 dairy worker samples through simple random sampling of study subjects that met our exclusion criteria (no antibiotic use within 3 months of baseline enrollment). All dairy worker samples were selected from workers on a single farm, and all identified as white Hispanic or Latino males (both the numbers of females working on the participating dairy farms and recruitment of females into the study was low). Selection of the 6 community control samples was done using simple random sampling among community participants who had no antibiotic use within 3 months of sample collection and baseline enrollment, and who covariate-matched our dairy workers on sex and ethnicity. The unbalanced sampling of each group was designed to over-sample dairy workers, as community control samples could be supplemented with additional healthy subjects’ metagenomics data from publicly available data (i.e., The Human Microbiome Project).

Study enrollment and baseline sample collection began in 2018 for these 16 participants. The collection of study samples occurred at least one year after the Food and Drug Administration completed implementation of the Guidance for Industry (GFI) no. 213 which restricted the use of antibiotics in animal agriculture for growth promotion purposes and transitioned medically important antibiotics used in drinking water and feed from over-the-counter status to Veterinary Feed Directive (VFD) or prescription status [36, 37].

### Sampling, shotgun metagenomic library preparation and sequencing

Stool samples were self-collected by participants using a stool specimen collection kit. Participants were instructed to store stool samples in their refrigerators and to return their stool samples within 24 hours of collection to study staff. Samples were stored at $-20^{\circ }$C by field staff at a partner study site for 1-6 months before before being packaged with dry ice and transported to the University of Washington for extraction and storage at $-20^{\circ }$C. DNA extraction was performed using the MoBio DNeasy PowerLyzer PowerSoil Kit (Qiagen) following manufacturer’s protocols, and quantification of the resulting DNA was conducted using the Quant-iT PicoGreen dsDNA Assay Kit (ThermoFisher/Invitrogen). Extracted DNA samples were packaged on dry ice and transported to the Fred Hutchinson Cancer Research Center for sequencing.

Sequencing libraries were prepared from 250pg gDNA with a quarter reaction workflow using the Nextera XT Library Prep Kit (Illumina, San Diego, CA) and 12 cycles of indexing PCR. Indexed libraries were pooled by volume and post-amplification cleanup was performed with 0.8X Agencourt AMPure XP beads (Beckman Coulter, Indianapolis, IN). The library pool size distribution was validated using the Agilent High Sensitivity D5000 ScreenTape run on an Agilent 4200 TapeStation (Agilent Technologies, Inc., Santa Clara, CA). Additional library QC and cluster optimization was performed using Life Technologies- Invitrogen QubitÂ® 2.0 Fluorometer (Life Technologies-Invitrogen, Carlsbad, CA, USA). The resulting libraries were sequenced on the Illumina HiSeq 2500 to generate paired-end 150nt sequences for each fragment. Image analysis and base calling were performed with Illumina Real Time Analysis software v1.18.66.3, followed by demultiplexing of dual-indexed reads, removal of adapters and primers, and generation of FASTQ files with bcl2fastq Conversion Software v1.8.4 [38].

### Profiling taxonomic composition

We performed profiling of the microbial composition of the metagenomic short reads of our dairy workers and community control samples with primers, adapters, and host sequences removed using MetaPhlAn3 v3.0.14 [39]. MetaPhlAn3 estimates relative abundances by mapping reads to a reference database of clade-specific marker genes from ChocoPhlAn v30 (published in January 2019) [39, 40]. MetaPhlAn3 performs this read mapping against marker genes using bowtie2 v2.3.5.1 [41, 42]. Default parameters were used when running MetaPhlAn3 with an additional flag -t rel_ab_w_read_stats for outputting relative abundances with estimated number of reads mapping to each clade.

### Metagenomic assembly and processing of contigs

We conducted *de novo* assembly and processing of contigs using anvi’o v6.2 [43]. anvi’o integrates a suite of bioinformatics tools for the processing, analyzing, and visualization of metagenomics, pangenomics, and phylogenomics studies. We used the anvi’o Snakemake [44] metagenomics workflow obtained from “anvi-run-workflow” [45] with “–workflow metagenomics” to conduct our metagenomic assembly and processing of contigs. Illumina-utils [46] was used to apply the guidelines of Minoche et al. [47] with a default parameter $p=0.75$ for quality filtering of reads based on Q-scores to trim and identify low quality reads. Details on the exact trimming and quality score filtering guidelines have been described in the literature [46, 47]. Further processing of the individual assemblies using anvi’o v6.2 included removal of human host reads using a GRCh38 reference, performing individual assembly of contigs for each sample metagenome [43] using MEGAHIT v1.2.9 [48], identifying open read frames (ORFs) using Prodigal v2.6.3 [49], predicting gene-level taxonomy using Centrifuge [50], functional annotation of genes using NCBI’s Clusters of Orthologous Groups (COGs) [51] and Pfams [52], searching for sequences using DIAMOND v0.9.14 [53], identifying single copy core genes (SCGs) using HMMER v3.3 [54] and built-in anvi’o Hidden Markov Model (HMM) profiles for bacteria and archaea, recruiting reads using bowtie2 v2.3.5.1 [41], and generating BAM files with samtools v1.10 [55]. Prediction of the approximate number of genomes in a metagenomic assembly using SCGs was done using the anvi’o script “anvi-display-contigs-stats”. Workflows using Snakemake with full parameter details can be found at the URL https://github.com/statdivlab/hdw_mgx_supplementary/.

### Metagenome annotation of virulence factors and antibiotic resistance genes

We used ABRicate v1.0.1 [56] to perform a mass screening of our *de novo* assembled gene calls identified from our assembled contigs for antibiotic resistance genes and virulence factor genes. ABRicate uses the Basic Local Alignment Search Tool (BLAST) [57] to annotate genes from a user-specified reference database. We used the Virulence Factor Database v6.0 [58] and the Comprehensive Antibiotic Resistance Database (CARD) v4.0 [59] as reference databases in our search. Genes were considered present in a given metagenome if they met conservative minimum thresholds of $90\%$ identity and $100\%$ coverage.

To compare gene abundances across samples we perform a normalization of gene abundances by creating a measure of relative gene abundance. Relative gene abundances were calculated within a metagenome by taking the mean coverage of a target ARG or VF gene divided by the sum of all mean coverages of all protein coding genes identified in a given metagenome. Here mean coverage indicates the average depth of coverage across a gene calculated by adding up the coverage of each nucleotide in a gene, and dividing by the length of the gene. ARG relative abundances were further aggregated by their antibiotic classes by summing the relative abundances of genes within each antibiotic class for each metagenome. We focused our analyses to antibiotic classes that were identified by the World Health Organization (WHO) as Critically Important Antibiotics (CIA) [60].

### Reconstruction of genomic context of ARGs

We used our results from ABRicate to extract ARG target sequences from each metagenomic assembly. These sequences were extracted using samtools [55] and were used as “query” sequences in our genomic context reconstruction analyses. ARG query sequences were used to produce query neighborhoods that reassociated unassembled or unbinned reads that are graph-adjacent to the query sequence. To prepare our raw metagenomic short reads for genomic context reconstruction, we removed adapters and quality trimmed the reads using fastp [61] before removing human host reads using bbduk [62] and the masked human k-mer data [63]. Using our quality trimmed and filtered short reads and our query sequences of interest, we constructed the genomic context of each query sequence using MetaCherchant [64]. MetaCherchant uses a de Bruijn graph assembly approach to build genomic context of query sequences. We used the “environment-finder” tool in parallel and set k-mer length to 31, minimum coverage to 5, and max radius to 1000. Taxonomic annotation of sequences corresponding to graph nodes was done using kraken2 v2.1.2 [65]. Taxonomic affiliation of genes was based on kraken2 annotations of surrounding graph nodes for a particular query sequence.

In order to understand whether the ARGs identified using ABRicate have historically been found on plasmids or microbial chromosomes, we cross-referenced the ARGs identified in our study with ARGs identified by the Resistance Gene Identifier (RGI) v5 [66]. The RGI integrates with the CARD database to predict AMR genes and their mutations in complete chromosome sequences, predicted genomic islands, complete plasmid sequences, and whole genome shotgun assemblies taken from National Center for Biotechnology Information (NCBI) databases. This is accomplished through prediction of ORFs using Prodigal [49], alignment to CARD reference sequences using either BLAST [57] or DIAMOND [53], and the use of either protein homolog or protein variant models. The results from RGI’s exhaustive search are maintained and updated for each antibiotic resistance gene catalog on the CARD database.

### Comparison with the Human Microbiome Project

To contextualize our study cohort, we also considered data from the Human Microbiome Project (HMP). Information on the study’s protocol, sampling, and sequencing procedures have been previously described [67–69]. Briefly, DNA was extracted using the Mo Bio PowerSoil DNA Isolation Kit and nucleic acid samples were quantified and checked for purity of the DNA with only samples with a minimum of 50-100 ng of DNA used. Libraries were prepared following a standard protocol from Illumina with modifications outlined in detail by the HMP study [69]. Processing of raw reads into contigs was conducted by the HMP study in the following steps: (1) sequencing of raw reads was performed using the Illumina GAIIx platform with 101bp paired-end reads, (2) all samples were screened for human contamination using NCBI’s BMTagger tool, with $\sim 49$% of reads targeted for removal as human, (3) samples underwent quality control assessments, including identification of outliers by mean contig and ORF density, human hits, rRNA hits and size, and (4) samples passing QC were assembled into contigs using IDBA-UD v1.1.0.

To identify samples, we utilized the curatedMetagenomicData (cMD) package [70], which provides curated and uniformly processed microbiome data. Raw sequences are downloaded by the cMD team and processed to produce taxonomic profiles using Metaphlan3 v3.0.0. We filtered for samples from the original HMP “Healthy Human Subjects” (HHS) study, and subset to first visits. In order to match our all-male cohort, we further subset to only male samples, resulting in 47 HMP samples for comparison. For taxonomic analyses we utilized abundance data from cMD, which used MetaPhlAn3 v3.0.0. Identification of antibiotic resistance genes and virulence factor genes was conducted using assembled contigs from the HMP portal, which were imported into anvi’o v6.2, where Prodigal v2.6.3 was used to identify ORFs. Annotation of ORFs for virulence factors and antibiotic resistance genes from the VFDB v6.0 and CARD v4.0 databases was conducted using ABRicate v1.0.1. We note that while information on occupation is not publicly available for the 47 HMP participants, the distribution of job types across the entire HMP cohort did not include any categories related to farm or animal work, and no more than 15% indicated an “other” category of occupation [71, 72]. Additionally, recruitment of individuals from the HMP focused on individuals in the general populations of two U.S. cities, St. Louis, Missouri and Houston, Texas [67]. We conclude that it is unlikely that any of the 47 HMP participants used in our study have occupational exposure to livestock farming. Information on race and ethnicity were not publicly available at the individual level for HMP participants, and thus, we were unable to adjust for race and ethnicity in our regression analyses.

### Statistical analyses

Differences in demographic and sequencing characteristics between groups were evaluated using two-sample t-tests allowing for heteroskedasticity (continuous variables) and $\chi ^2$ tests for independence (categorical variables). To test for differential abundance (at the species and phyla level) between dairy workers and community controls, we used radEmu v1.2.0 [73] with species coverages as the response and an indicator for dairy work (the predictor of interest) and age (a potential confounder) as predictors. We conducted robust score tests and applied a false discovery rate (FDR) correction using the qvalue v2.26.0 [74] package. Secondary analysis of species differential abundance that included both data from this study as well as HMP data were performed similarly, but with the inclusion of an indicator for the participant being from the HMP study, thus adjusting for cohort and batch effects. radEmu accounts for differential sequencing depth, and is robust to the differential detectability of bacteria taxa and unobserved species in samples. By estimating fold-changes, radEmu addresses limitations of analyzing bacterial proportions from high-throughput sequencing data [75].

For our $\alpha$-diversity analysis, we estimated the Shannon Diversity Index (SDI) using the DivNet v0.4.0 [76] model applied to MetaPhlAn3 relative abundances using an identity design matrix. We compared the SDI of dairy worker and community control metagenomes with betta [77], using an indicator for dairy work and age as predictors. Our $\beta$-diversity analysis estimated Bray-Curtis dissimilarities using MetaPhlAn3 relative abundances, and tested for differences in $\beta$-diversity using the testBetaDiversity function as implemented in DivNet with an indicator for dairy work as a predictor. This test was performed using a bootstrapped pseudo-F test with 10,000 bootstrap iterations. DivNet accounts for uncertainty in estimating SDI and Bray-Curtis dissimilarity arising from sample-to-sample variation and taxon co-occurrence. We estimated gene richness using breakaway v4.8.2 [78] and compared gene richness between dairy worker and community control metagenomes using function betta as integrated in geneshot v0.6.2 [79, 80], using an indicator for dairy work as a predictor. In addition to our gene richness analysis, we investigated species richness using single-copy core genes and breakaway and betta, testing for differences in species richness between dairy worker and community control metagenomes. Estimating gene and genome richness with breakaway accounts for unequal sequencing effort across samples by estimating unobserved species, and comparing estimated richness using betta accounts for uncertainty and heteroskedasticity in total richness estimates.

Relative abundances of ARGs were calculated using the mean coverage for a given target gene divided by the sum of all mean coverages of all the protein coding genes identified in a given metagenome. We applied a centered log-ratio (CLR) transformation on our relative abundance data (pseudocount $1 \times 10^{-15}$), filtering out genes in the database that had zero abundance across all samples. Differential abundance testing of antibiotic resistance genes in dairy workers compared to community controls was performed using linear regressions with CLR-transformed relative abundances as the response and an indicator for dairy work (the predictor of interest) and age (as a potential confounder) as predictors. We use robust standard errors implemented in rigr v1.0.4 [81]. This approach targets a similar estimand to radEmu, but can be parallelized for large-scale analysis [75]. Testing for differential presence of virulence factor genes and ARGs between dairy workers and community controls was performed using happi v0.8.7 [82], adjusting for depth as a quality variable. To conduct gene enrichment testing, we used happi’s likelihood ratio testing (LRT) procedure with default parameters, setting the number of permutations used to 1000 and contamination probability $\varepsilon = 0.05$. Comparisons of gene presence (response) between dairy workers and community controls using happi included an indicator for dairy work and age as predictors. For differential gene enrichment testing between our study cohort and the HMP participants we used indicators for dairy work and study cohort (which adjusts for batch/cohort variability) as well as age and BMI in the models. We performed FDR corrections for multiple comparisons for all tests using the qvalue v2.26.0 R package for q-value estimation. All statistical analyses were conducted using R v4.1.2.

### Results

#### Study description

At baseline enrollment, the dairy worker cohort averaged 10 years (SD 5.2) of experience in the dairy industry. Compared to community controls, dairy workers were notably younger, with a mean age of 38.40 years compared to 49.50 years for controls (t-test $p=0.06$, see Table 1). Both groups had similar proportions of current smokers (67% for controls vs. 70% for dairy workers, $\chi ^2=1$). Furthermore, there were no statistically significant differences between dairy workers and community controls in terms of body mass index (BMI) or consumption habits of alcohol, dairy products, vegetables, eggs, beef, chicken, lamb, and fish (Table 1) at the $5\%$ level. All community controls reported occupations as field workers in non-animal agriculture at the time of sample collection and study enrollment.

**Table 1:** Demographic and behavioral characteristics of community controls (CC) and dairy workers (DW) from the Healthy Dairy Worker (HDW) cohort and participants from the Human Microbiome Project (HMP). *p*-values correspond to tests of equality of mean (continuous variables) or distribution (categorical variables) across dairy workers and community controls. Limited metadata (at the level of individuals) was made publicly available from the HMP participants, and has been denoted as n/a (data not available) where appropriate

	Community (N=6)	Dairy (N=10)	HMP (N=47)	*p*-value (DW vs. CC)
**Age (years)**				
Mean (SD)	49.5 (10.7)	38.4 (8.0)	$$\text {26.5 (4.7)}^{**}$$	0.06
**Body Mass Index**				
18.5-24.9	0 (0%)	2 (20.0%)	$\text {33 70.2\%)}^{\mathrm{a}}$	0.41
25-29.9	4 (66.7%)	4 (40.0%)	$\text {10 (21.3\%)}^{\mathrm{a}}$	
30.0+	2 (33.3%)	4 (40.0%)	$\text {4 (8.5\%)}^{\mathrm{a}}$	
**Current Smoker**				
No	4 (66.7%)	7 (70.0%)	n/a	1
Yes	2 (33.3%)	3 (30.0%)		
**Alcohol Consumption**				
Never	1 (16.7%)	2 (20.0%)	n/a	0.15
Rarely <1/week	4 (66.7%)	2 (20.0%)		
1-7 times/week	1 (16.7%)	6 (60.0%)		
**Dairy Consumption**				
Rarely <1/week	1 (16.7%)	2 (20.0%)	n/a	1
1-7 times/week	5 (83.3%)	8 (80.0%)		
**Vegetable Consumption**				
Rarely <1/week	0 (0%)	1 (10.0%)	n/a	1
1-7 times/week	6 (100%)	9 (90.0%)		
**Eggs Consumption**				
Rarely <1/week	1 (16.7%)	2 (20.0%)	n/a	1
1-7 times/week	5 (83.3%)	8 (80.0%)		
**Beef Consumption**				
Rarely <1/week	3 (50.0%)	1 (10.0%)	n/a	0.23
1-7 times/week	3 (50.0%)	9 (90.0%)		
**Chicken Consumption**				
Rarely <1/week	1 (16.7%)	0 (0%)	n/a	0.79
1-7 times/week	5 (83.3%)	10 (100%)		
**Lamb Consumption**				
Never	5 (83.3%)	7 (70.0%)	n/a	1
Rarely <1/week	1 (16.7%)	3 (30.0%)		
**Fish Consumption**				
Never	0 (0%)	1 (10.0%)	n/a	0.59
Rarely <1/week	3 (50.0%)	3 (30.0%)		
1-7 times/week	3 (50.0%)	6 (60.0%)		
**Occupation**				
field worker	6 (100%)	0 (0%)	n/a	-
dairy worker	0 (0%)	10 (100%)		

$$^{**}$$two-sample t-test *p*-values $$< 0.001$$ for HMP compared to HDW

$^{\mathrm{a}}\chi ^{2}<0.001$ for HMP compared to HDW

In our study cohort, the total number of paired reads in metagenomes ranged from 18–33 million per sample, with community control samples yielding significantly more paired reads compared to dairy worker samples ($27.1 \times 10^6$ vs. $22.8 \times 10^6$, t-test $p=0.04$, Table 2). An average of 88.3% (SD 1.1%) of paired reads corresponding to a mean of $24 \times 10^6$ paired reads (SD $4.3 \times 10^6$) (Table 2, Supplementary Table S1) passed quality filtering and trimming. Host reads made up 0.05%-1.55% of samples. Removal of host reads resulted in an average of $24.2 \times 10^6$ (SD $2.9 \times 10^6$) paired reads in community control samples and $20.1 \times 10^6$ (SD $4.0 \times 10^6$) paired reads in dairy worker samples used for our analyses.

**Table 2:** Summary statistics related to sequencing data for the dairy worker (DW) and community controls (CC) from the Healthy Dairy Worker (HDW) cohort and participants from the Human Microbiome Project (HMP). $\text {M} = \times 10^6$, $\text {k} = \times 10^3$

	Community (N=6)	Dairy (N=10)	HMP (N=47)	*p*-value (DW vs. CC)
**No. of pairs sequenced**				
Mean (SD)	27M (3.1M)	22.8M (4.3M)	n/a	0.04
**Total pairs post-filtering**				
Mean (SD)	24.2M (2.9M)	20.1M (4.0M)	n/a	0.03
**Total pairs post-host removal**				
Mean (SD)	24.2M (2.9M)	20.1M (4.0M)	$$\text {111M (26.4M)}^{**}$$	0.03
**No. of contigs**				
Mean (SD)	58.6k (8k)	43.4k (16k)	$$\text {105k (49k)}^{**}$$	0.02
**No. genes (Prodigal)**				
Mean (SD)	240k (26k)	181k (59k)	$\text {221k (95k)}^{\ddagger }$	0.02

$$^{**}$$two sample t-test $$p < 0.001$$ for HMP compared to HDW

$^{\ddagger }$two sample t-test $p=0.37$ for HMP compared to HDW

To provide context for our study cohort, we compared our study participants with data from 47 healthy males participating in the Human Microbiome Project (HMP). Information available for these individuals was limited to age, sex, and BMI (Table 1). On average, HMP participants were notably younger than our study cohort (26.5 years vs. 42.6 years, t-test $p<0.001$, see Table 1) and had lower BMI ($\chi ^2 < 0.001$). Sequencing depths for the HMP males ranged from 21–239 million paired reads per sample. The average number of paired reads for the HMP cohort after quality filtering and host sequence removal was significantly higher than that of our study cohort ($111 \times 10^6$ vs. $21.6 \times 10^6$, t-test $p<0.001$, Table 2).

### Taxonomic profiling of dairy worker and community control metagenomes

**Fig. 1:**
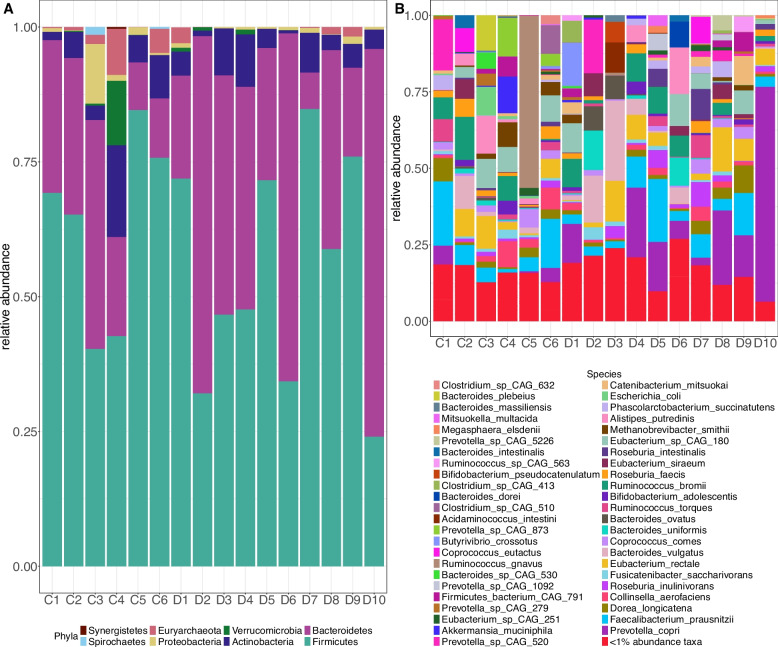
Stacked barplots of relative abundances show the most abundant phyla (**A**) and species (**B**) within each metagenome. At the phylum-level (**A**), Firmicutes, Bacteroidetes, and Actinobacteria are the most abundant phyla across all samples. At the species-level (**B**), the 5 most abundant and prevalent species across community control and dairy worker metagenomes were *F. prausnitzii*, *E. rectale*, *P. copri*, and *Eubacterium* sp. CAG-180. Species with relative abundances less than 1% were grouped together. There was insufficient evidence to suggest major differences in the taxonomic composition of dairy worker metagenomes compared to community controls

The 16 metagenomic samples were composed of 9 distinct phyla: Firmicutes, Bacteroidetes, Actinobacteria, Verrucomicrobia, Proteobacteria, Euryarchaeota, Spirochaetes, unclassified Eukaryota and Synergistetes. Of these phyla, Firmicutes, Bacteroidetes, and Actinobacteria were the 3 most abundant phyla found across all samples (Fig. 1A). The large representation of Firmicutes, Bacteroidetes, and Actinobacteria reflected similar community compositions observed in healthy subjects from the Human Microbiome Project [68]. We also note that while the majority of the phyla identified are from the domain Bacteria, we observed organisms from the domains Archaea (Euryarchaeota) and Eukaryota as well. We detected Euryarchaeota organisms in 5 dairy worker and 6 community control samples and unclassified Eukaryota organisms in low abundances in 2 dairy worker samples from our study. We conducted differential abundance testing comparing phylum-level differences between dairy workers and community controls, controlling for age, and found no significant differences at the 5% FDR level in phylum abundances (Supplementary Table S2).

At the species-level, we identified 272 different species across the 16 metagenomes. The most prevalent bacteria species observed were *Prevotella copri*, *Faecalibacterium prausnitzii*, *Eubacterium rectale*, *Ruminococcus bromii*, and *Bacteroides vulgatus* (Fig. 1, right). These five species have been previously shown to be highly abundant organisms found in healthy human gut microflora [83–87]. Differential abundance testing revealed no statistically significant differences in the abundances of these five organisms between occupational groups (adjusting for age) at an FDR of 5% (Supplementary Table S3). Abundance patterns for the five species with the most extreme test statistics (Supplementary Table S3) showed higher abundances of *Ruminoccocus lactaris* ($\hat{\beta }=3.20$, $q=0.19$) in dairy workers and higher abundances of *Methanobrevibacter smithii* ($\hat{\beta }=-1.12$, $q=0.19$), *Methanosphaera stadtmanae* ($\hat{\beta }=-11.20$, $q=0.19$), *Clostridium* sp. CAG:167 ($\hat{\beta }=-4.21$, $q=0.19$), and *Prevotella* sp. CAG:873 ($\hat{\beta }=-8.10$, $q=0.19$) in community controls. We additionally conducted differential abundance testing comparing dairy workers and non-dairy workers with the inclusion of metagenomic data from 47 HMP male subjects with no evidence of disease. A comparison with and without the inclusion of HMP cohort showed similar effect sizes (Pearson $\hat{\rho }=0.71$), but some variation in *p*-values (Pearson $\hat{\rho }=0.55$) (Supplementary Figure S1).

We further investigated differences in the community structures of dairy worker and community control metagenomes by examining differences in $\alpha -$ and $\beta -$ diversities. A comparison of the species-level $\alpha -$diversity using Shannon diversity showed no significant difference in the $\alpha -$diversity of dairy worker metagenomes compared to community control metagenomes ($\hat{\alpha }_{\text {DW}} - \hat{\alpha }_{\text {CC}} = -0.19$, betta $p=0.24$). Similarly, a comparison of differences in the community composition ($\beta -$diversity) of dairy worker and community control metagenomes using the Bray-Curtis dissimilarity metric showed no evidence of differences in the true group centroids at the $5\%$ level (DivNet $p=0.40$) (Supplementary Figure S2). We additionally analyzed differences in gene-level richness (the number of unique genes) between dairy worker and community control metagenomes using geneshot [79, 80] and breakaway [78] to estimate the gene-level richness of each sample, finding significantly lower gene-level richness in dairy worker metagenomes compared to community control metagenomes ($\hat{C}_{\text {DW}} -\hat{C}_{\text {CC}} \approx -2.0 \times 10^{5}$, breakaway $p=0.003$, [77]). To contextualize this finding, we also estimated the species richness in each metagenome using single-copy core genes [43], finding that on average there were 55 fewer species in dairy worker metagenomes compared to community control metagenomes, but that this difference was not statistically significant at the 5% level (breakaway $p=0.31$).

### Identification of virulence factor genes

**Fig. 2:**
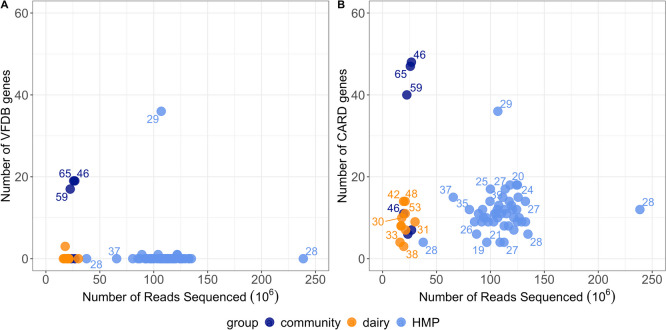
For each metagenome, we compare the sequencing depth with the number of identified (**A**) VFDB genes and (**B**) CARD genes. Ages (years) of each subject have been labeled. Samples with deeper sequencing had higher numbers of identified genes from the CARD and VFDB databases and higher numbers of estimated genomes. Within the community control group, 3 samples had the highest number of identified CARD genes out of all samples studied, whereas the remaining 3 community control samples within the community control group appeared to be indistinguishable from dairy workers in the number of identified CARD genes. The number of CARD and VFDB genes identified in our study cohort appeared to be similar in range to the number of CARD and VFDB genes identified in the HMP HHS cohort despite higher sequencing depths on average per sample in the HMP study cohort

Through mass screening of contigs across our 16 metagenomes using the Virulence Factor Database (VFDB) [58], we identified 37 different virulence factor genes across 4 samples (3 community controls and 1 dairy worker; Supplementary Table S4). We found that samples with the highest number of identified Virulence Factor Database (VFDB) genes were also those with higher sequencing depth (Fig. 2, right). On average, community control samples had higher number of virulence factor genes identified than dairy workers (mean $= 9.2$, sd 10.1 vs. mean $= 0.3$, sd 0.9, p = 0.08). Using happi [82], which accounts for unequal sequencing effort, we tested for differential enrichment of virulence factor genes between dairy worker and community control metagenomes, adjusting for age. No virulence factor genes were significantly enriched between dairy worker and community control metagenomes at the 5% FDR level (Supplementary Table S5). We note that 3 community control metagenomes had higher numbers of identified virulence factor genes compared to samples of similar sequencing depth.

For comparison, we also considered the number of virulence factor genes identified in the HMP HHS cohort. When comparing the male subjects from the HMP cohort to our all-male dairy worker cohort, we found that the HMP males had a range of 4-36 virulence factor genes, which was higher than the range of 0-19 virulence factor genes found in our study metagenomes (Fig. 2A). Testing for differential enrichment of virulence factor genes between dairy workers and non dairy workers, adjusting for HMP cohort membership, age and BMI and accounting for sequencing depth did not show evidence of significantly enriched virulence factors genes between dairy workers and non dairy workers at the 5% FDR level (Supplementary Table S6).

### Identification and taxonomic associations of antimicrobial resistance genes (ARGs)

Screening of the 16 metagenomes using the Comprehensive Antibiotic Resistance Database (CARD) identified 85 distinct ARGs across the 16 metagenomes, conferring resistance to at least 17 different antibiotic classes (Supplementary Figure S3; Supplementary Table S7). On average, a higher number of ARGs were identified in community control metagenomes compared to dairy worker metagenomes (mean $= 26.5$, sd 20.5 vs. mean $= 8.5$, sd 3.7, p = 0.09) (Fig. 2B). However, differences in the number of ARGs identified may be due, in part, to differences in sequencing depth, as metagenome samples with the highest number of ARGs identified also had higher numbers of sequenced reads (Fig. 2B). We therefore used happi to test for differences in the enrichment of ARGs between dairy worker and community control metagenomes, while accounting for differences in sequencing depth and adjusting for age. No ARGs were differentially enriched at 5% FDR, but the following ARGs had the largest magnitude test statistics: *sat4* (happi LRT $\chi ^2 = 0.001, q=0.10$) a plasmid-mediated streptothricin acetyltransferase and streptothricin resistant determinant, *tet*(W) (happi LRT $\chi ^2 = 0.05,q=1$) a tetracycline resistance gene associated with both conjugative and non-conjugative DNA, and *emrB* (happi LRT $\chi ^2 = 0.14,q=1$) a translocase gene in the emrB-TolC efflux protein in *Escherichia coli* (Supplementary Table S8). The 3 community metagenomes that we found to have higher numbers of virulence factor genes identified in their metagenomes also had higher numbers of ARGs identified compared to other metagenomes of similar sequencing depth. To contextualize our study cohort, we compared the number of ARGs found in our study metagenomes with the number of ARGs identified in the HMP HHS. Overall, the range of ARGs identified in our study cohort (3-48 ARGs) was similar to the range of ARGs identified in the HMP cohort (4-36 ARGs) (Fig. 2B). Testing for differential enrichment of ARGs between dairy workers and non dairy workers while adjusting for HMP study membership, age and BMI and accounting for sequencing depth did not show evidence of significantly enriched ARGs between dairy workers compared to non dairy workers at the 5% FDR level (Supplementary Table S9).

**Fig. 3:**
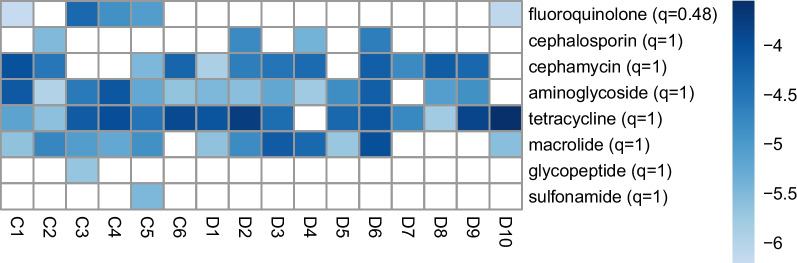
We identified ARGs from 8 antibiotic classes (rows) listed as critically important to human medicine by the WHO. $\log _{10}$ transformed relative abundances of antibiotic resistance genes grouped by these antibiotic classes are colored from lower (light blue) to higher (darker blue) relative abundances in each metagenome. Antibiotic resistance classes (rows) have been ordered by ascending q-values. White squares denote undetected antibiotic resistance genes. Visual inspection displays patterns of increased abundance of tetracycline resistance genes and macrolide resistance genes in dairy worker metagenomes. Additionally, cephamycin resistance genes had a higher occurrence in dairy workers as these genes were identified in 90% of dairy worker samples compared to 67% of community control samples

We further focused our analyses to ARGs conferring resistance to antibiotic classes considered critically important to human medicine by the World Health Organization (WHO) [60]. Across our study metagenomes, we identified 37 different ARGs conferring resistance to 8 antibiotic classes described in the WHO’s list of Critically Important Antimicrobials (CIA): aminoglycosides, fluoroquinolones, macrolides, tetracyclines, cephalosporins, cephamycins, glycopeptides, and sulfonamides (Fig. 3). The most frequently occurring types of antibiotic resistance genes found across the 16 metagenomes were genes that typically confer resistance to tetracyclines ($$n = 15$$), aminoglycosides ($$n = 14$$), cephamycins ($$n = 13$$), and macrolides ($$n = 12$$) (Fig. 3). Genes that commonly confer tetracycline resistance appeared to dominate the resistomes of both dairy workers and community controls with 11 distinct tetracycline resistance genes identified across 15 of our study metagenomes. We compared relative abundances of genes aggregated by antibiotic class between both groups and found that, after adjusting for age, dairy workers’ metagenomes had higher centered log-ratio transformed relative abundances of tetracycline ($$\hat{\beta }_{DW} = 2.7$$, $$q=1$$), cephamycin ($$\hat{\beta }_{DW} = 5.4$$, $$q=1$$), macrolide ($$\hat{\beta }_{DW} = 3.9$$, $$q=1)$$, sulfonamide ($$\hat{\beta }_{DW} = 1.6$$, $$q=1)$$, and cephalosporin ($$\hat{\beta }_{DW} = 4.1$$, $$q=1)$$ resistance genes than community controls’ metagenomes (Fig. 3; Supplementary Table S10); however, these differences were not significant at the 5% FDR. Similarly, the lower CLR-transformed relative abundance of fluoroquinolone ($$\hat{\beta }_{DW} = -19.2$$, $$q=0.5$$), aminoglycoside ($$\hat{\beta }_{DW} = -3.11$$, $$q=1$$), and glycopeptide ($$\hat{\beta }_{DW} = -5.1$$, $$q=1$$) resistance genes in dairy workers’ metagenomes compared to community controls’ metagenomes was also not significant at an FDR of 5%.

To understand whether there were differences in taxonomic affiliation of ARGs between groups, we assessed the taxonomic context of tetracycline and beta-lactam resistance genes. We identified 6 different genes (*cblA*-1, *cfx*A2, *cfx*A3, *cfx*A4, *cfx*A5, *cfx*A6) that encode for beta-lactamases and confer resistance to beta-lactam antibiotics. Additional details on the presence of each beta-lactam resistance gene in each of our study metagenomes are found in Supplementary Table S7. These 6 beta-lactam genes have typically been identified on the chromosomes of *Bacteroides* spp. [66]. Taxonomic annotation of the genomic context of these genes in dairy worker and community control metagenomes confirmed their association with organisms from the phylum Bacteroidetes such as *Prevotella copri*, *Bacteroides fragilis*, and *Bacteroides uniformis*. Additionally, we observed no differences in taxonomic affiliation of these beta-lactam genes between dairy workers and community controls (Supplementary Table S11).

We identified 9 *tet* genes (efflux genes: *tet*(B), *tet*(G), *tet*(40); and ribosomal genes: *tet*(M), *tet*(O), *tet*(Q), *tet*(W), *tet*(W/N/W), and *tet*(32)) that encode for efflux pumps or ribosomal protection proteins conferring resistance to tetracycline antibiotics. These genes have normally been associated with plasmids [66], which are small, extra-chromosomal DNA molecules that facilitate genetic sharing between and within species [88], but can also be found in chromosomes. Taxonomic annotation of the assembly graphs for these tetracycline resistance genes demonstrated affiliation of these genes with a variety of both commensal (e.g., *Lawsonia intracellularis*, *Ligilactobacillus animalis*, *Trueperella pyogenes*, *Schaalia turicensis*, and *Faecalibacterium prausnitzii*) and pathogenic (e.g., *Campylobacter* spp., *Clostridium* spp.) bacteria. Full annotations of these ARGs to affiliated bacterial organisms can be found in Supplementary Table S11. Finally, while these tetracycline resistance genes were affiliated with a wide range of commensal and pathogenic bacteria, we found no differences in the taxonomic context of tetracycline resistance genes identified in community controls compared to dairy workers.

### Discussion

Using shotgun metagenomics sequencing, we investigated differences in taxonomy, diversity, and the presence of genes (especially ARGs and virulence factors) between dairy workers and community controls’ gut microbiomes. The use of shotgun metagenomics data allowed us to circumvent some of the limitations of amplicon sequencing, and enabled us to investigate abundance and presence of a variety of genes as well as their taxonomic context. While the results of our investigation revealed no statistically significant differences at the 5% FDR level in the taxonomic composition, antibiotic resistance and virulence factor gene carriage, and relative abundances of ARGs, we observed several patterns for further investigation including greater abundance of tetracycline resistance genes and higher occurrence of cephamycin resistance genes in dairy workers’ metagenomes; evidence of commensal organism association with tetracycline resistance genes; and lower gene richness and genome diversity in dairy workers’ metagenomes.

Previous metagenomic studies of livestock workers in China and Europe have found increased abundance and carriage of antibiotic resistance genes in individuals occupationally exposed to animal farming environments, raising concerns that these environments could be hotspots for antibiotic resistance and zoonotic disease emergence [27–29, 89, 90]. Cross sectional studies of pig farmers and slaughterhouse workers in the Netherlands ($n_{\text {workers}}=70$, $n_{\text {controls}}=46$) [28] and China ($n_{\text {workers}}=4$, $n_{\text {controls}} = 5$) [90] found that the resistomes of these animal workers were dominated by tetracyclines, aminoglycosides, beta-lactam and macrolide resistance genes. Another cross-sectional study of live poultry market workers in China found higher abundance of ARGs, lower Shannon diversity, and greater enrichment of beta-lactam and lincosamide resistance genes in these workers compared to controls ($n_{\text {workers}}=18$, $n_{\text {controls}}=18$) [89], and a longitudinal study of veterinary students with exposure to swine farms observed similar patterns of increased total abundance of ARGs and increased abundances of beta-lactam, aminoglycoside, and tetracycline resistance genes within 3 months $n=14$ [27].

Contrary to these previous studies of livestock workers, we found no significant difference in the abundance of ARGs between dairy workers and community controls, though we did observe patterns of greater abundance of tetracycline resistant genes in dairy workers’ metagenomes that was directionally consistent with findings in these previous farm studies [27–29, 89]. In addition, we found more frequent occurrences of cephamycin (beta-lactam) resistant genes identified in the dairy worker population compared to community controls. These patterns are interesting to highlight since tetracyclines are commonly administered on dairy farms for treating gastrointestinal and respiratory diseases in dairy cows [91] and beta-lactam antibiotics such as ceftiofur are frequently used to treat metritis, a common post-partum uterine inflammatory disease [92]. It is also worth noting that the patterns observed in our study reflect the potential impacts of occupational exposure to livestock farming without the use of antibiotics for growth promotion, as the samples used in this metagenomics study were collected at least one year after the full implementation of the FDA’s GFI no. 213 policy banning the use of antibiotics for growth promotion purposes.

Our study also highlighted the potential for commensal organisms to serve as ARG reservoirs for pathogenic bacteria. By reconstructing the genomic context of each antibiotic resistance gene then taxonomically annotating this context, we were able to confirm the association of chromosome-mediated ARGs (e.g., *cblA*-1, *cfx*A2, *cfx*A3, *cfx*A4, *cfx*A5, *cfx*A6) with previously recognized carriers of these genes (e.g., *Bacteroides* spp.) [66]. With the same approach applied to primarily plasmid-mediated ARGs (e.g., *tet*(B), *tet*(G), *tet*(W/N/W), *tet*(32), *tet*(M), *tet*(O), *tet*(Q), and *tet*(W)), we found that resistance genes were associated with both commensal and pathogenic organisms. These observations suggest the potential for sharing of ARGs between commensal organisms and pathogens through conjugation. Furthermore, our results corroborate findings from a recent study that compared ARGs identified in 1,354 culture commensal strains and 45,403 pathogen strains from the human gut and found evidence of 64,188 shared ARGs that mapped to 5,931 mobile genetic elements [93]. Some of the mobile genetic elements identified [93] had also been previously identified in data from ruminant guts, soil, and other human body sites [93]. While commensal organisms may serve as ARG reservoirs for pathogenic bacteria, they may also assist in preventing pathogenic invasion through indirect (enhancement of host immune defenses) and direct (competition of nutrients and niche) mechanisms [8–11]. Further research is needed to better understand the complex dynamic that commensal organisms balance in promoting both pathogen resistance and antibiotic resistance emergence.

Our results also demonstrated evidence of lower average gene richness (and some evidence of lower genome diversity) in dairy workers, even after accounting for differential sequencing depth. Lower gene richness has been associated with increased intestinal inflammation and metabolic disorders [79, 94, 95]. A common occupational hazard facing dairy workers is inhalation of dusts and aerosols containing endotoxins or other proinflammatory substances that can result in airway inflammation and decreased pulmonary function [96–98]. Several studies have proposed a gut-lung axis linking pulmonary inflammation to intestinal inflammation based on epidemiological and clinical observations of the co-occurrence of these diseases [99–101]. There is therefore the possibility that the lower gene richness observed in dairy workers points towards increased intestinal inflammation linked to possible increased airway inflammation from exposure to aerosols and endotoxins. Further investigation to explore the possibility of increased intestinal and airway inflammation of this cohort is warranted.

Our study had several limitations. The most significant limitation was its small sample size, and therefore relatively low power to reject false null hypotheses. Corroborating our findings, especially those regarding patterns of greater tetracycline and cephamycin resistance gene in our dairy cohort, with a larger sample size is desirable. Another major limitation of our study was the comparability of the community controls with the dairy workers. The community controls in our metagenomic study occupationally identified as field workers in non-animal agriculture industries, and agricultural and dairy workers both experience occupational exposure to animal manure (e.g., as fertilizer) and antibiotics (e.g., streptomycin and oxytetracycline are commonly sprayed to control fire blight disease in Eastern Washington [102, 103]). Similar occupational exposures in the dairy workers and controls may reduce the effect sizes of group differences compared to comparisons of dairy workers and non-agricultural workers (e.g., office workers).

That our cohort is comprised of exclusively Hispanic or Latino males limits the generalizability of our findings. The American Community Survey, conducted by the US Department of Agriculture in 2021 [104], indicates that livestock workers across the US are 22% female and only 48% non-Hispanic Whites. The results observed from our study therefore may not be broadly generalizable to livestock worker cohorts.

We additionally note the higher average age of community controls in our cohort compared to dairy workers, with 3 community controls having both higher ages and the highest number of identified ARGs and virulence factors. Antibiotic resistance genes have been previously shown to have an age-related cumulative effect, with older age groups harboring higher abundances of ARGs [105]. To mitigate this, our gene enrichment and gene abundance analyses adjusted for age as a potential confounder (note also that all our analyses adjust for unequal sequencing depth across samples). Our analyses didn’t reveal any statistically significant differences in ARG presence between groups of the same age who differ in their dairy worker status. A secondary analysis that supplemented our cohort with the Human Microbiome Project cohort (to contextualize the dairy workers with an alternative control group) also did not suggest that there are differences in ARG presence between groups of the same age, BMI and study cohort who differ in their dairy worker status. One limitation of this secondary analysis is that the HMP data was not processed alongside our study’s samples, and technical artifacts inducing falsely significant results are a substantial concern when pooling data across studies in general. A comparison of the extraction, sequencing and data processing protocols between the HMP data and our study cohort show notable differences in sequencing platforms (HMP used an Illumina GAIIx platform with 101bp paired-end reads compared to our study’s use of an Illumina HiSeq 2500 with 150bp paired-end reads), quality control and filtering methods (HMP removed duplicate and low-quality reads while also filtering out outliers by mean contig and ORF density, human hits, rRNA hits and size whereas our study employed a Q-score based algorithm to identify and filter out low quality reads), host removal tools used (HMP used BMTagger while our study used bowtie2), and *de novo* assemblers used (HMP used IDBA-UD compared to our study’s use of MEGAHIT). However, as our ARG enrichment results were null (both within our study and when supplemented with HMP data), technical artifacts do not appear to be driving significantly different associations. Additionally, adjusting for study using an indicator for the participant being from the HMP cohort in our analyses addresses the potential differential detection of microbial taxa and genes across the two studies that might arise due to differences in extraction, library preparation protocols, and bioinformatics processing.

Another limitation of this secondary analysis using the HMP data is the potential misclassification of occupation in the HMP participants. In the absence of publicly available data on occupation, we relied on previous studies and limited published information on the distribution of job types across the HMP cohort. The HMP’s 2012 phase one study participants of HHS had an over-representation of students with job categories reported in research, healthcare, education, sales, clerical/administrative, management, and an “other” category [72]. The “other” category made up no more than 15% of the responses collected. Additionally, recruitment of individuals from the HMP was conducted in two urban areas [67]. As many dairy workers live isolated in rural areas, having poor access to public transportation and many lacking personal vehicles [106], it is unlikely that any of the 47 HMP participants used in our study had occupational exposure to livestock farming. We further note that there is only limited publicly available demographic data on the HMP participants, and therefore we could only adjust for age and BMI as potential confounders. Thus, while there is a possibility of unmeasured confounding in our study, we can rule out the risk of confounding bias as the cause of significant associations between ARG presence and dairy work.

Finally, while cross-sectional studies can be advantageous for conducting cost-effective comparisons of populations, they only capture differences between groups at a single time point. Therefore, our study cannot provide information about long-term changes to the microbiome that are induced by occupational exposure to livestock farming. We also note that our choice to use shotgun metagenomics to studying antibiotic resistance limits our conclusions to genotypic potential for resistance, and not phenotypic resistance. Complementary future work includes pairing shotgun sequencing with phenotypic resistance profiles (e.g., using culture-based approaches).

### Conclusions

In this study of occupational exposure to dairy farming, we observed no significant differences in antibiotic resistant gene or virulence factor presence in dairy workers compared to controls, but several patterns warranting further investigation, including greater abundance of tetracycline resistance genes and higher occurrence of cephamycin resistance genes in dairy workers’ metagenomes; evidence of commensal organism association with tetracycline resistance genes; and lower average gene richness in dairy workers’ metagenomes. This work demonstrates the depth and scope of utilizing shotgun metagenomics to investigate microbiomes and resistomes, and provides a foundation for further investigations into the impact of exposure to zoonotic pathogens, antibiotic resistant organisms, and ARGs on the microbiomes and resistomes of livestock workers.

## Supplementary information

Supplementary Material 1.

Supplementary Material 2.

## Data Availability

The data supporting the conclusions of this article along with code for reproducing our results are made available at https://github.com/statdivlab/hdw_mgx_supplementary. The sequencing data has been made available on the Sequence Read Archive under PRJNA971196.

## Abbreviations

ARG(s): Antibiotic Resistance Gene(s)
WHO: World Health Organization
HDW: Healthy Dairy Worker Study
VF: Virulence Factors
VFDB: Virulence Factor Database
CARD: Comprehensive Antibiotic Resistance Database
CIA: Critically Important Antimicrobials
LRT: Likelihood Ratio Test
CLR: Centered Log-Ratio
SCG(s): Single Copy Gene(s)
DNA: Deoxyribonucleic acid
sd: standard deviation
