# Supplementary figures and images for: A cross-sectional comparison of gut metagenomes between dairy workers and community controls
Source: BMC Genomics. 2024 Jul 20;25:708. doi: 10.1186/s12864-024-10562-1 (PMC11626760; doi:10.1186/s12864-024-10562-1)

## Supplementary Figures

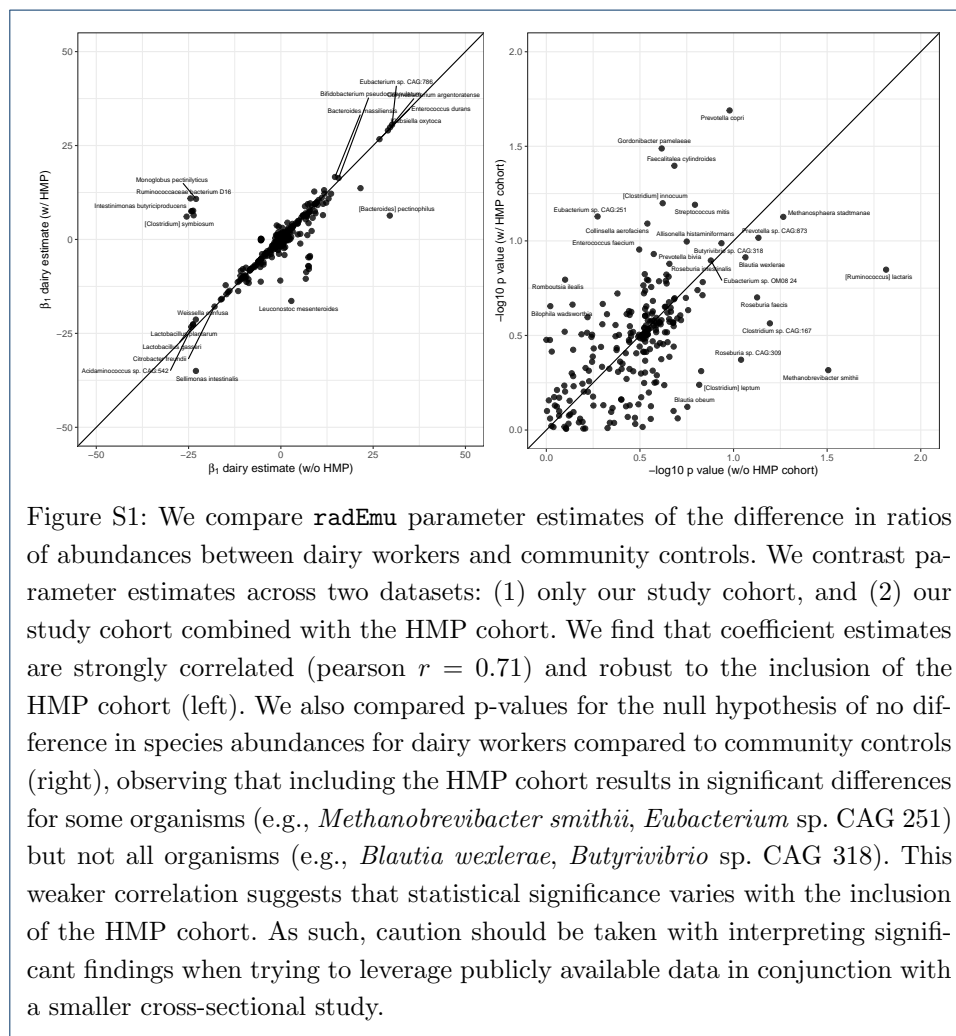

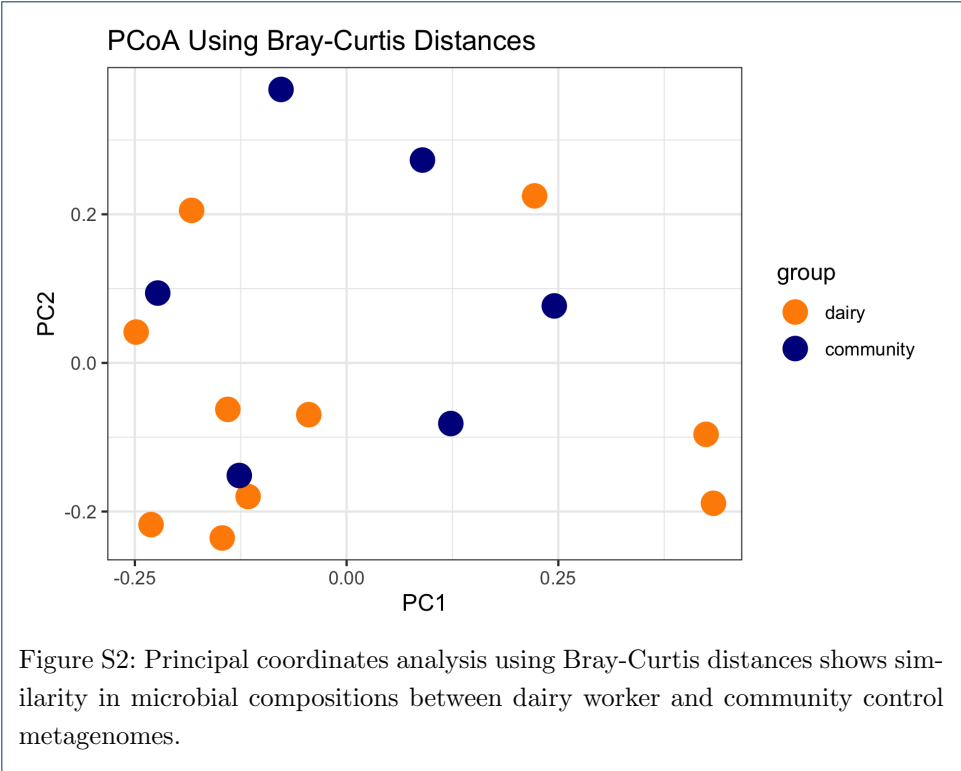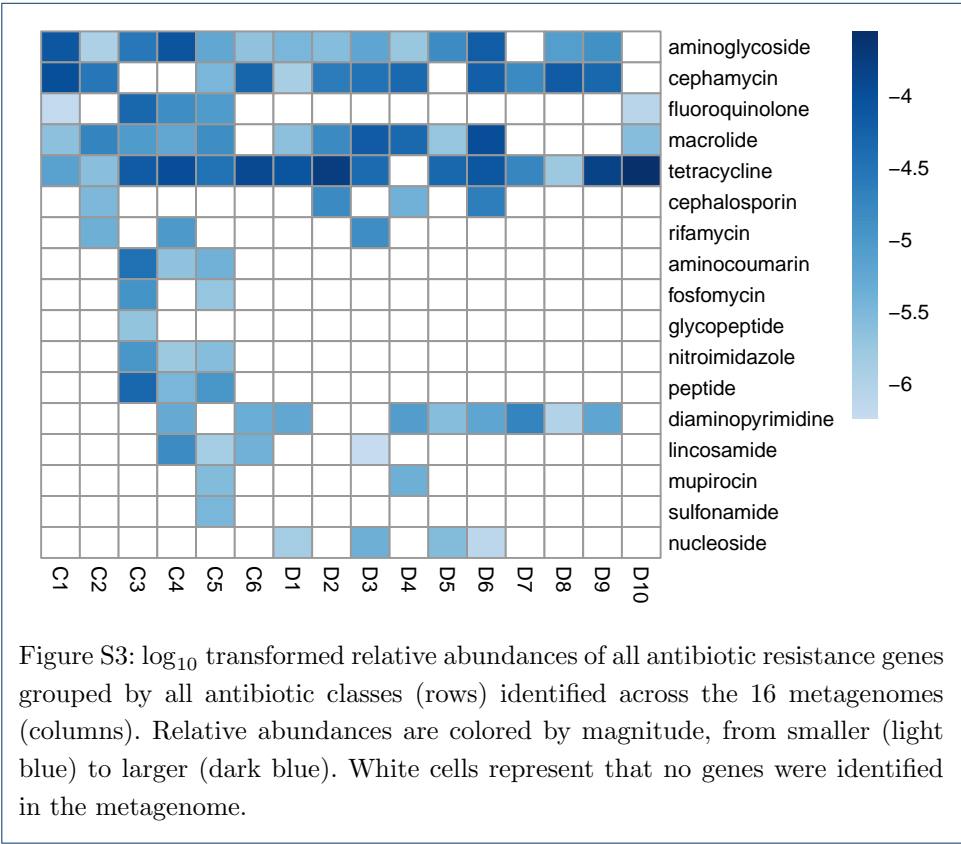

Supplement: Supplementary file 2 — Supplementary Material 2. [file 12864_2024_10562_MOESM2_ESM.pdf]
